# Epidemiology of Psychiatric Disorders in Iranian Children and Adolescents (IRCAP) and Its Relationship with Social Capital, Life Style and Parents' Personality Disorders: Study Protocol

**Published:** 2017-01

**Authors:** Mohammad Reza Mohammadi, Nastaran Ahmadi, Koorosh Kamali, Ali Khaleghi, Ameneh Ahmadi

**Affiliations:** 1 Psychiatry and Psychology Research Center, Tehran University of Medical Sciences, Tehran, Iran.; 2 Yazd Cardiovascular Research Center, Shahid Sadoughi University of Medical Sciences, Yazd, Iran.; 3 Zanjan University of Medical Sciences, Zanjan, Iran.

**Keywords:** *Adolescents*, *Children*, *Psychiatric Disorders*, *Social Capital*

## Abstract

**Objective: **We aimed at designing a cross sectional study to investigate the prevalence of psychiatric disorders in Iranian children and adolescents (IRCAP) and to determine its relationship with social capital, life style, and parents' personality disorders.

**Method:** This cross sectional study was a national project implemented in all provinces of Iran. In this community-based study, using ‎multistage cluster sampling method, we selected 1000 children and adolescents aged 6 to 18 years in each province. The total sample size reached to 31 000. ‎We randomly collected 170 blocks. Then, of each cluster head, we selected 6 cases including 3 cases of each gender in ‎different age groups (6- 9 years, 10- 14 years, and 15- 18 years). The clinical psychologists instructed the participants to complete the Persian version of Kiddie-Sads-‎Present and Lifetime Version (K-SADS-PL). In addition, demographic data (gender, age, education, parent education, and economic situation) and information on lifestyle, social capital, and parents' personality disorders were obtained from the participants.

**Discussion: **IRCAP study presents a protocol for an epidemiological survey on the first estimates for the prevalence of psychiatric disorders in children and adolescents across the country. This large body of data, on a range of individual behavioural and emotional items and scores, allows us to compare the rates and patterns of deviance between urban and rural places of residence in 31 provinces of Iran with non Iranian samples surveyed with the same measures.

The latest research revealed that almost 20% to 49% of children and adolescents suffer from some form of psychiatric disorders. Thus, there is a strong need to better understand the prevalence of mental disorders and related factors in children and adolescents in Iran. Lack of mental health services is a priority on the agenda (1). Conducting studies on psychiatric disorders in a community is necessary to provide effective psychiatric services (2).

In 2013, Mohammadi et al. investigated the epidemiology of psychological problems in 5171 adolescents aged 6 to 17 years in 5 provinces of Tehran, Khorasan Razavi, Isfahan, East Azerbaijan, and Fars in Iran. 

They found that conduct problems had the highest prevalence of psychological problems and social problems the lowest prevalence in the 5 provinces. Moreover, they found that males had less emotional problems than females. In addition, they obtained no significant difference between 12 to 14 and 15 to 17 year old adolescents or between middle and high school graduates in psychological problems. In the present study, the prevalence of psychological problems in adolescents in the urban population in Fars province was higher than expected (3).

Studies on prevalence of child and adolescent psychiatric disorders in different parts of the world present diverse reports. The prevalence rates of psychopathology for mental health problems of children and adolescents in the general population was 10% in Denmark, 7% in rural Brazil and Norway, 10% in Britain and Denmark, and up to 15% in Russia and Bangladesh (4-10).

In Iran, one prevalence study indicated that approximately 17.9% of 6 to 11 year-old children in Tehran suffer from psychiatric disorders (11).

However, a considerable discrepancy has been found between prevalence rates and the number of children being treated through childhood and adolescence. Factors associated with the development of psychopathological disorders include age and gender, location, socioeconomic markers, and family conditions (12). 

In the study by H.-U. Wittchen et al. on the prevalence of mental disorders and psychosocial impairments in adolescents and young adults, it was found that substance disorders were the most frequent (lifetime 17±7%; 12-month 11±4%), with abuse being considerably more frequent than dependence; and the prevalence of other mental disorders was about 27±5% (12-month, 17±5%). Depressive disorders (16±8%) were more frequent than anxiety disorders (14±4%), and eating disorders (3±0%) and threshold somatoform disorders (1±2%) were rare disorders (13).

In the study by Yung Shin kim et al. in 2011 on the prevalence of autism spectrum disorders in a total population sample, the prevalence of autism spectrum disorders found to be 2.64% (14).

Moharreri et al. conducted a study in 2009, entitled: “The Epidemiological Survey of Psychiatric Disorders in Children and Adolescents of Mashhad”. In their study, 2012 children and adolescents aged 6 to 18 years were selected from different areas of Mashhad using clustering method. Parents and adolescents filled out the SDQ. After analyzing the self-report form of the SDQ, it was found that 34% of the participants had psychological problems, and this rate was 67.7% in the parent form of the SDQ (15).

A study was conducted by Kathleen Ries Merikangas et al. entitled:” The Prevalence and Treatment of Mental Disorders Among US Children”. In their study, the sample included 3042 participants aged 8 to 15 years from the cross sectional surveys conducted from 2001 to 2004 done by NHANES. The Twelve-month prevalence rates based on the Diagnostic and Statistical Manual of Mental Disorders, Fourth Edition, were 8.6% for attention-deficit/ hyperactivity disorder, 3.7% for mood disorders, 2.1% for conduct disorder, 0.7% for panic disorder or generalized anxiety disorder, and 0.1% for eating disorders their sample (16).

A study was conducted by Cohen et al. on the relationship between negative life events and psychological disorders and the roles of positive life events and received and perceived social support in moderating this relationship. In their study, the cross sectional analyses, but not the prospective analyses, provided some support for the stress-buffering (interaction) effects of positive events (17).

The study by Costa-Requena et al. examined perceived social support in Spanish cancer outpatients with psychiatric disorders and found that the perception of social support received by the patient increased by psychopharmacology treatment. Considering the importance of perceived social support for the psychological wellbeing of patients, healthcare professionals could provide support to normalize the distress of cancer patients (18).

The study by Karlidere et al. revealed that males had less social support and emotional distress and females had less sexual function problems. However, satisfactory social support might decrease the emotional symptoms of both genders (19).

***Overall Aims of the Study***

The present study aimed at investigating the prevalence of psychiatric disorders in Iranian children and adolescents (IRCAP) and its relationship with social capital, life style, and parents' personality disorders.

***Hypothesis***

- There is a relationship between psychiatric disorders and social capital in Iranian children and adolescents.
- There is a relationship between psychiatric disorders and life style in Iranian children and adolescents.
- There is a relationship between psychiatric disorders and parents' personality disorders in Iranian children and adolescents.

***The Study Objectives***

- To determine the prevalence of psychiatric disorders in children and adolescents
- To determine the frequency of psychiatric disorders in children and adolescents according to demographic data (gender, age, education, parent education, and economic situation)
- To determine the assessment of social capital in children and adolescents
- To determine the assessment of different lifestyles in children and adolescents
- To determine the level of social capital in children and adolescents
- To determine the prevalence of parents personality disorders
- To determine the relationship between psychiatric disorders and lifestyle in children and adolescents
- To determine the relationship between psychiatric disorders and parents personality disorders in children and adolescents
- To determine the relationship between psychiatric disorders and social capital in children and adolescents

***Applied*** ***Objectives***

- To provide statistics on the prevalence of psychiatric disorders for health policy makers
- To identify children and adolescents at high risk of psychiatric disorders for primary prevention
- To conduct workshops of life skills for positive cases (In these workshops self-awareness, empathy, effective communication, interpersonal relations, decision making, problem solving, creative thinking, critical thinking, problem solving ability, and ability to cope with stress are taught.)
- To conduct workshops of appropriate life style for children and adolescents

## Materials and Methods

***Study Design***

This was an **analytical cross sectional** study and a national project implemented in all provinces of Iran. Moreover, the National Institute for Medical Research Development (NIMAD) financially supported this study. 

The principal applicant has conducted large-scale surveys of psychiatric disorders and has particular expertise in utilizing the instruments used in this proposal. Preliminary work was undertaken 4 years prior to conducting this study. First, the screening questionnaires were translated into Farsi, piloted with families, and back-translated into English by professional translators. This procedure was repeated several times before the final version could be obtained. Second, a pilot epidemiological investigation was conducted on a large (N = 2000) sample of Iranian children in Teheran schools by the principal investigator, providing the applicants with the experience of such investigations. Third, an epidemiological investigation was done by the principal investigator to investigate the epidemiology of psychological problems in 5171 adolescents aged 6 to 17 years in 5 provinces of Tehran, Khorasan Razavi, Isfahan, East Azerbaijan, and Fars. Fourth, the principal investigator and his colleagues reported the test-retest reliability and the inter-rater reliability of the Persian version of K-SADS, and found the sensitivity and specificity of the Persian version to be high (20). The main format of this protocol was adapted from Yazd Health Study Protocol (21).

***Sampling***

In a community-based study, 1000 children and adolescents aged 6-18 years were selected from each province by ‎multistage cluster sampling method (cluster and stratified random sampling). ‎Then, 170 blocks were randomly collected. Of each cluster head, 6 cases were selected, with 3 cases of each gender in ‎different age groups (6- 9 years, 10 -14 years, and 15- 18 years). The blocks were selected randomly according to postal.

***Inclusion and Exclusion Criteria***

Inclusion ‎criteria were as follow: Being an Iranian citizen (In each province, people who resided at least one year in that province could participate in the project.), and age range of 6 to18 years. Children and ‎adolescents with severe physical illness were excluded.‎

***Data Collection***

The clinical psychologists instructed the participants to complete the Persian version of Kiddie-Sads-‎Present and Lifetime Version (K-SADS-PL). Trained psychologists referred to the children's home and interviewed them using the K-SADS-PL. The time required to complete the K-SADS ‎was about 30 to 40 minutes. In addition, demographic data (gender, age, education, parent education, and economic situation), information about lifestyle, social capital, and parents' personality disorders were obtained. ‎ 

***Procedures***

***1. The Site:***

Iran is the 16th largest country in the world, and has a total population of 78.47 Million inhabitants (71.2% in urban areas, and 28.8% in rural areas); of the total population of Iran, 85% are over the age of 6 and literate; and unemployment rate is around 11% among adults. The population is ethnically diverse with large groups from Turkish, Kurdish, Lorish, Baluchi and Arabic origins. The religion is Islam (98.8%), and Farsi is the official language (The only language used for writing in administrations and the main language used for teaching in schools). Iran has 31 provinces, with 104 114 schools (primary and secondary) that provide education to 16 million pupils aged 7 to 18 years.

***2. Selection of Study Areas:***

The IRCAP survey was conducted in 31 provinces of Iran, including the capital, and provided an opportunity to compare the provinces, which differ in background characteristics such as ethnicity mix, culture, and economic wealth, allowing the detection of fine-tuned variations in the rates of individual behavioural and emotional problems in children; this might call for differential service provision. The sample was selected from the all the 31 provinces of the country. In each province, measures were administered in Farsi. Within each area, the sample was selected in 2 zones to provide a contrast between urban and rural places of residence. 

***3. Overall Study Design:***

In the screening and diagnostic stage, a random sample of the population of children aged 6 to 18 years was surveyed with K- SADS-PL measures of known reliability and validity. A multi-informant approach was used and the parents were asked to complete the screening questionnaires simultaneously and independently and the youths themselves were asked to complete the questionnaires if they were 11 years or older. This large body of data, on a range of individual behavioural and emotional items and scores, allows us to compare the rates and patterns of deviance within and between the 31 provinces of Iran with non-Iranian samples surveyed with the same measures. 

***4. Selection of the Participants:***

IRCAP project is a national project implemented in all provinces of Iran. This project, using a semi- structured interview, K-SADS-PL, focused on the study of psychiatric disorders among 31 000 children and adolescents aged 6 to 18 years. The sample size was calculated to provide an appropriate estimation in provinces. Assuming a prevalence of psychiatric disorders of equal to 0.3 and type one error of 0.05, and accepted error of 0.05, the sample size was calculated to be equal to 825 for each province. We suggested the design effect for cluster sampling as 1.2, so the final sample size in each province increased to 990 (1000). The total sample size reached to 31 000, and 170 blocks (6 samples in each) were selected in each province. The multistage cluster sampling was considered for this study. In each province, in addition to the main city, rural places were selected randomly as a cluster sampling. In the next step, the blocks in provinces were selected randomly according to postal code. We had samples from urban and rural areas in provinces proportionally.

In addition to investigating the psychiatric disorders, Millon Clinical Multiaxial Inventory, Social Capital Questionnaire, and Life Style Questionnaire were used.

***Scales***

***Kiddie-SADS-Present and Lifetime Version (K-SADS-PL):***

KSADS- PL, the Schedule for Affective Disorders and Schizophrenia for School-Age Children/Present and Lifetime Version, is a semi-structured psychiatric interview that is based on DSM-IV criteria. It contains 5 diagnostic groups: (1) affective disorders including depression disorders [major depression, dysthymia] and mania, hypomania; (2) psychotic disorders; (3) anxiety disorders including social phobia, agoraphobia, specific phobia, obsessive- compulsive disorder, separation anxiety disorder, generalized anxiety disorder, panic disorder, and posttraumatic stress disorder; (4) disruptive behavioral disorders including ADHD, conduct disorder, oppositional defiant disorder; and (5) substance abuse, tic disorders, eating disorders, and elimination disorders (enuresis/encopresis) (22). 

The interview starts with questions about basic demographics. Moreover, information about presenting complaints and prior psychiatric problems are also obtained (23). 

Ghanizadeh et al. have reported the reliability of the Persian version of this questionnaire to be 0.81 and the inter-rater reliability to be 0.69 using test-retest. The sensitivity and the specificity of the Persian version of K-SADS found to be high (24). In a study of Polanczyk et al. kappa coefficients were 0.93 (p<0.001) for affective disorders, 0.9 (p<0.001) for anxiety disorders, and 0.94 (p<0.001) for ADHD and disruptive behavior disorders. The present study assessed the interrater agreement for K-SADS (25).

***Millon Clinical Multiaxial Inventory - Third Edition (MCMI-III)*** **:**

The Millon Clinical Multiaxial Inventory - Third Edition (MCMI-III) is the most recent edition of the inventory, and it is a psychological tool for assessing personality traits and psychopathology including specific psychiatric disorders outlined in the DSM-IV. It is intended for adults (18 and older), with at least a 5^th^- grade reading level. The MCMI was specifically developed and standardized for clinical populations (ie, patients in clinical settings or people with existing mental health problems) (26). 

The MCMI-III was published in 1994 and the reflected revisions were made in the DSM-IV. Total numbers of scales in MCMI-III are 14 personality scales, 10 clinical syndrome scales, and 5 correction scales. The third edition contains 175 true-false questions, taking approximately 20 to 25 minutes to complete. The inventory is almost self-administered (27). 

Blais et al. (2003) found that the MCMI-III Avoidant Scale is reliable (r =.89). They demonstrated appropriate convergent and divergent validity with other self-report measures. The MCMI-III Anxiety scale also showed adequate reliability (r =.78) (28). Dyer (1997) concluded that the MCMI-III has content validity against the DSM-IV, which is superior to any other major personality instrument (29).

**Social Capital Questionnaire (Nahapyt and Ghoshal,** ***1998):***

Social capital questionnaire of "Nahapyt and Ghushal" (1998) contains 28 questions that deals with cognitive social capital, communication, and structural. This questionnaire contains 7 subscales, which are as follow: networks, trust, cooperation, mutual understanding, relationships, values, and commitment (30). 

Content validity was used to determine the validity of the questionnaires. In addition, professors' corrective opinions were applied in this field. In this research, the Cronbach’s alpha coefficient was used to assess reliability. The rate of social capital questionnaire reliability coefficient was 85/0, showing an optimal reliability of this questionnaire: the reliability coefficient of the cognitive social capital component was 89/0, that of the relation social capital was 9/0 and that of the structural social capital was 8/0 (31).

***Life Style Questionnaire (LSQ):***

This questionnaire was made by Lali et al. (32) in Iran; its validity was confirmed through factor analysis and its reliability through internal consistency method; the Cronbach’s alpha ranged from 0.79 to 0.89 for different subscales (33). The LSQ contains 70 items in 10 subscales including physical health, sports and fitness, weight management and nutrition, disease prevention, mental health, spiritual health, social health, avoidance of drugs, alcohol and opiates, accident prevention, and environmental health. All items are responded on a four-point Likert scale scoring in the range from 0 (= never) to 3 (= always). The higher the score, the better the lifestyle (32).

***Ethics***

Consent was obtained from children and adolescents (Consent was completed for participants younger than 15 years by their parents and for participants aged 15 to 18 years by parents or by the adolescents themselves.). Information about children and adolescents and their families was kept confidential.

If children or adolescents were diagnosed with a psychiatric disorder, the child and adolescent psychiatrist, who collaborated on the IRCAP project, treated them out of charge. However, if the participants or their parents did not wish to use the free treatment, then, they were referred to other child and adolescent psychiatrists. 

The national institute for medical research development (NIMAD) supported this study (the ethics code of IR.NIMAD.REC.1395.001).

***Analysis***

Data were entered into the SPSS 16. To determine the frequency of psychiatric disorders and lifestyle in children and adolescents, we used descriptive analysis and 95% ‎confidence interval. A p value of <0.05 was considered statistically significant. We used one-‎way ANOVA to test the significant differences of the disorders according to gender, age, education, parent education, and economic situation.

In the present study, correlation was used to examine the following relationships: the relationship between the frequency of psychiatric disorders and parents' personality disorders, between the frequency of psychiatric disorders and social capital, and between the frequency of psychiatric disorders and life style in children and adolescents. Moreover, regression was used to control the confounding variables.

## Discussion

Recently, Iran has undergone important social and economic changes. With a population of 78.47 million (50% below the age of 25, and 18 million between the ages of 7 and 18), a substantial number of children and adolescents might suffer from emotional or behavioral disorders that could have substantial implications for health services. 

Child psychiatry is still in its infancy in Iran and the existing studies are often too simple in their methods to yield the information that is really needed. Such countries do, nevertheless, have particularly strong concerns about child mental health and strong needs for epidemiological surveys. Rapid sociocultural, political, and economic changes may affect the lifestyles of communities and families, and influence the physical and psychological wellbeing of children. A recent review of studies examining the rates of behavioral and emotional disorders in children living in Iran suggests that children living in big cities in the country have rates of problems (20% to 40%) as high as, or higher than those living in developed countries. However, we need more valid and detailed information about the mental health of Iranian children and adolescents to improve our youth’s health policymaking. 

The present study was a protocol for an epidemiological survey that provided the first estimates for the prevalence of specific child psychiatric disorders in a large, representative community sample of Iranian youths. These data will be compared to estimates from other countries and will provide a baseline against which further future estimates could be compared to detect time trends. The survey will allow for an estimation of service needs when planning services is under way. Variations of rates and patterns of behavioural deviance in the country will give possible clues on risk factors for different subcultures and will guide a more precise services planning. Survey data for Iranian children will be compared with those from other countries (both developed and underdeveloped) surveyed with the same methodology. The health professionals who work with children in Iran will have access to a large database on normative behaviours and emotions of a large representative group of children and adolescents; this knowledge is necessary to calibrate assessments and interventions in clinical settings and to facilitate research.

## Limitations

The IRCAP study was the first epidemiological survey of psychiatric disorders in a large sample of children and adolescents living in Iran. 

This study was conducted in the provincial capitals. In the case of facilities, project could also be conducted in other cities of the province; and in that case, a more accurate prevalence of psychiatric disorders in children and adolescents could be estimated.
